# Two new species of *Parapharyngodon* parasites of *Sceloporus pyrocephalus*, with a key to the species found in Mexico (Nematoda, Pharyngodonidae)

**DOI:** 10.3897/zookeys.559.6842

**Published:** 2016-02-03

**Authors:** Edgar Uriel Garduño-Montes de Oca, Rosario Mata-López, Virginia León-Règagnon

**Affiliations:** 1 Departamento de Biología Evolutiva, Facultad de Ciencias, UNAM. C.P. 04510, Coyoacán, D. F., México; 2 Estación de Biología Chamela, Instituto de Biología, Universidad Nacional Autónoma de México, San Patricio, Jalisco, 48980, México

**Keywords:** Helminth, Enteric nematode, Reptile, Lacertilia, Phrynosomatidae, Michoacán, Guerrero

## Abstract

Two new species of *Parapharyngodon* collected from the intestine of the Mexican boulder spiny lizard *Sceloporus pyrocephalus* are described. This study increases to 49 the number of valid species assigned to *Parapharyngodon* worldwide, 11 of them distributed in Mexico. Males of the two new species share the presence of four pairs of caudal papillae, an anterior echinate cloacal lip and the presence of lateral alae; however, both differ from each other in lateral alae extension and echinate cloacal anterior lip morphology. Females of both species have a prebulbar uterus and eggs shell punctuate with pores, characteristics shared with few other species of *Parapharyngodon*. Both new species differ from other congeneric species in the papillar arrangement, the anterior cloacal lip morphology, the lateral alae extension and total length/spicule ratio. A taxonomic key for the species of *Parapharyngodon* distributed in Mexico is provided.

## Introduction

Mexico has a species-rich reptile fauna, with 864 species (8.7% of the worldwide total); 57% of them are endemic (Flores-Villela and García-Vázquez 2014). Although Mexico has a long tradition of helminthological and herpetological research, there are large gaps in the knowledge of the species diversity of helminths associated with these vertebrates (Pérez-Ponce de León et al. 2011). *Sceloporus* Wiegmann is a reptile genus distributed in the Americas; it inhabits a wide range of environments, and it is the most representative lizard taxon of the Mexican herpetofauna with 92 of 97 species that composed the genus, with the majority of them endemic (Flores-Villela 1993). *Sceloporus pyrocephalus* Cope is an oviparous endemic lizard to Mexico. It is associated with streams and rivers within tropical deciduous and semi-deciduous forest, and it is distributed from the Southwestern Pacific coast of Jalisco and Colima to Michoacán, Guerrero, South-western Mexico State, and Southern Morelos (Uetz and Hošek 2013). There are scarce studies on this phrynosomatid lizard; the majority of these are focused in taxonomy, conservation, and reproductive research (Ramírez-Bautista and Olvera-Becerril 2004, Calisi et al. 2007, Leaché 2010).

Recently, the validity of species of *Parapharyngodon* Chatterji, 1933 was reviewed (Velarde-Aguilar et al. 2015, Bursey and Goldberg 2015). In accordance with these authors, of the 78 *Parapharyngodon* species assigned to this genus, only 47 have been properly described, and recognized as valid species. Nine of these species are distributed in the Panamanian realm, seven in Oriental, seven in Palearctic, six in Neotropical, five in Afrotropical, five in Nearctic, five in Saharo-Arabian, one Australian, one in Sino-Japanese realm, and one species in Madagascan region. In this paper, two new species of *Parapharyngodon* parasites of *Sceloporus pyrocephalus* collected from Michoacán and Guerrero states, Mexico, are described, increasing the basic knowledge about helminths of Mexican lizards.

## Materials and methods

During the breeding season months of June-July in 2003, 2004 and 2005, 16 *Sceloporus pyrocephalus* were captured (under the collection permit SEMARNAT FAUT-0056 issued to VLR) by noosing or hand in ten different locations (seven from Michoacán and three from Guerrero states, Mexico, Table 1). Hosts were killed by an intraperitoneal injection of sodium pentobarbital overdose. The mouth, peritoneal cavity and all internal organs were examined for helminths with the use of stereoscope. Nematodes found were counted, fixed in hot 4% formaldehyde solution and stored in alcohol 70%. For morphological study, specimens were cleared in glycerin-alcohol 70% solution at 1:1 ratio, and mounted on temporary slides for examination under a light microscopy. Original drawings were made with an Olympus BX53 microscope equipped with camera lucida. For scanning electron microscopy (SEM), worms were dehydrated through ethanol series, dried with a K850 Critical Point Drier (Emitech, Ashford, England), coated with gold using a Q150R Modular Coatin System (Quórum, Ashford, England), and examined in a Hitachi S-2460N (Hitachi, Tokyo, Japan) and SU1015 SEM (Hitachi) SEM. Measurements are provided in millimeters, including the range, followed by average and standard deviation, and the sample size. Host specimens collected were deposited in the Herpethology Collection of the Museo de Zoología, Facultad de Ciencias (MZFC), UNAM, and helminths were deposited in the Colección Nacional de Helmintos (CNHE), Instituto de Biología, UNAM.

**Table 1.** Sampling sites for *Sceloporus pyrocephalus* analyzed in this study.

Locality (reviewed host) geographic coordinates	Nematode species (specimens obtained)	Collecting date
Michoacán		
Aquila (1) 18.5911 N, 103.5667 W	*Parapharyngodon ayotzinapaensis* sp. n. (17)	07/2003
Arteaga (3) 18.6468 N, 101.9684 W	*Parapharyngodon ayotzinapaensis* sp. n. (2) *Parapharyngodon tikuinii* sp. n. (5)	07/2005
La Huacana (1) 18.6734 N, 101.9951 W	*Parapharyngodon tikuinii* sp. n. (1)	07/2005
Tepalcatepec (1) 19.0758 N, 102.8936 W	*Parapharyngodon tikuinii* sp. n. (1)	06/2004
Álvaro Obregón (1) 19.0386 N, 102.9744 W	*Parapharyngodon tikuinii* sp. n. (1)	06/2004
Buenavista (2) 19.1766 N, 102.6635 W	*Parapharyngodon tikuinii* sp. n. (5)	07/2005
Apatzingan (1) 19.1247 N, 102.4014 W	*Parapharyngodon tikuinii* sp. n. (1)	07/2003
Gabriel Zamora (3) 19.1764 N, 102.0633 W	*Parapharyngodon ayotzinapaensis* sp. n. (8) *Parapharyngodon tikuinii* sp. n. (4)	06/2004
Guerrero		
Tecpan de Galeana (2) 17.2967 N, 101.0467 W	*Parapharyngodon ayotzinapaensis* sp. n. (3) *Parapharyngodon tikuinii* sp. n. (1)	07/2004
El Patio (1) 17.177 N, 100.5953 W	*Parapharyngodon ayotzinapaensis* sp. n. (12)	07/2005

## Results

### Family Pharyngodonidae Travassos, 1920 Genus *Parapharyngodon* Chatterji, 1933

#### Parapharyngodon ayotzinapaensis sp. n.

Taxon classificationAnimaliaOxyuridaPharyngodonidae

http://zoobank.org/E06EF8E0-3F05-4A60-A599-4DAEDC81353A

Figs 1A–E ; 2A–H

##### Type material.

Holotype, male, CNHE 9432. Allotype, female, CNHE 9433. Paratypes (4 males, 7 females), CNHE 9434-9438.

##### Etymology.

The species is named in honor of the 43 missing students from “Escuela Normal Rural Raúl Isidro Burgos” in Ayotzinapa, Guerrero, Mexico; in solidarity with their families and the Mexican people.

##### Diagnosis.

Robust, small and white fusiform nematodes, males smaller than females. Cuticle with prominent transverse striations along the whole body except tail. Triangular oral opening surrounded by three simple lips in males and bilobed in females, in both sexes ventrolateral lips have an amphid each one, in females it is located on the dorsal lobe. Within buccal cavity, both sexes have three transverse plates, bilobed in males and complete in females. Esophageal bulb with sclerotized apparatus. Excretory pore evident, it is located at level of posterior edge of esophageal bulb. A vesicular body surrounds excretory duct. Males with lateral alae covering the last third of body, females lacking lateral alae. Males without caudal alae. Four pairs of caudal papillae. Caudal filament sub-terminal and directed dorsally in males. Females with a conical posterior end. Vulva located at middle region. Eggs no shown alae, with a punctuated shell and subpolar operculum, embryo in early stage of cleavage.

##### Description of male.

**Figure 1.**
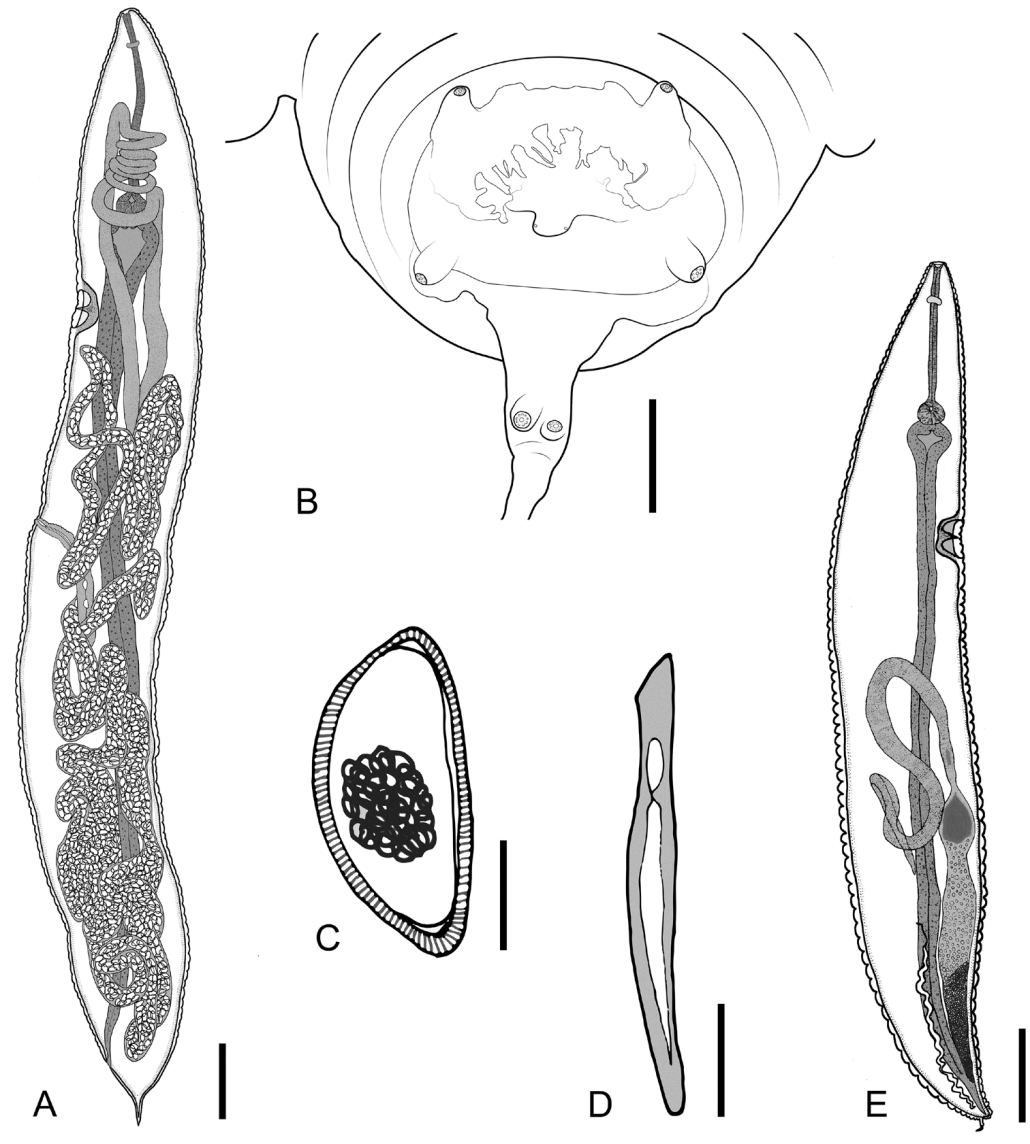
*Parapharyngodon ayotzinapaensis* sp. n. **A** Gravid female, entire, lateral view **B** Male, caudal end ventral view **C** Egg, lateral view **D** Spicule **E** Male, entire, lateral view. Scale bars = (**A, E**) 0.5 mm, (**B**) 0.025 mm, (**C, D**) 0.025 mm.

**Figure 2.**
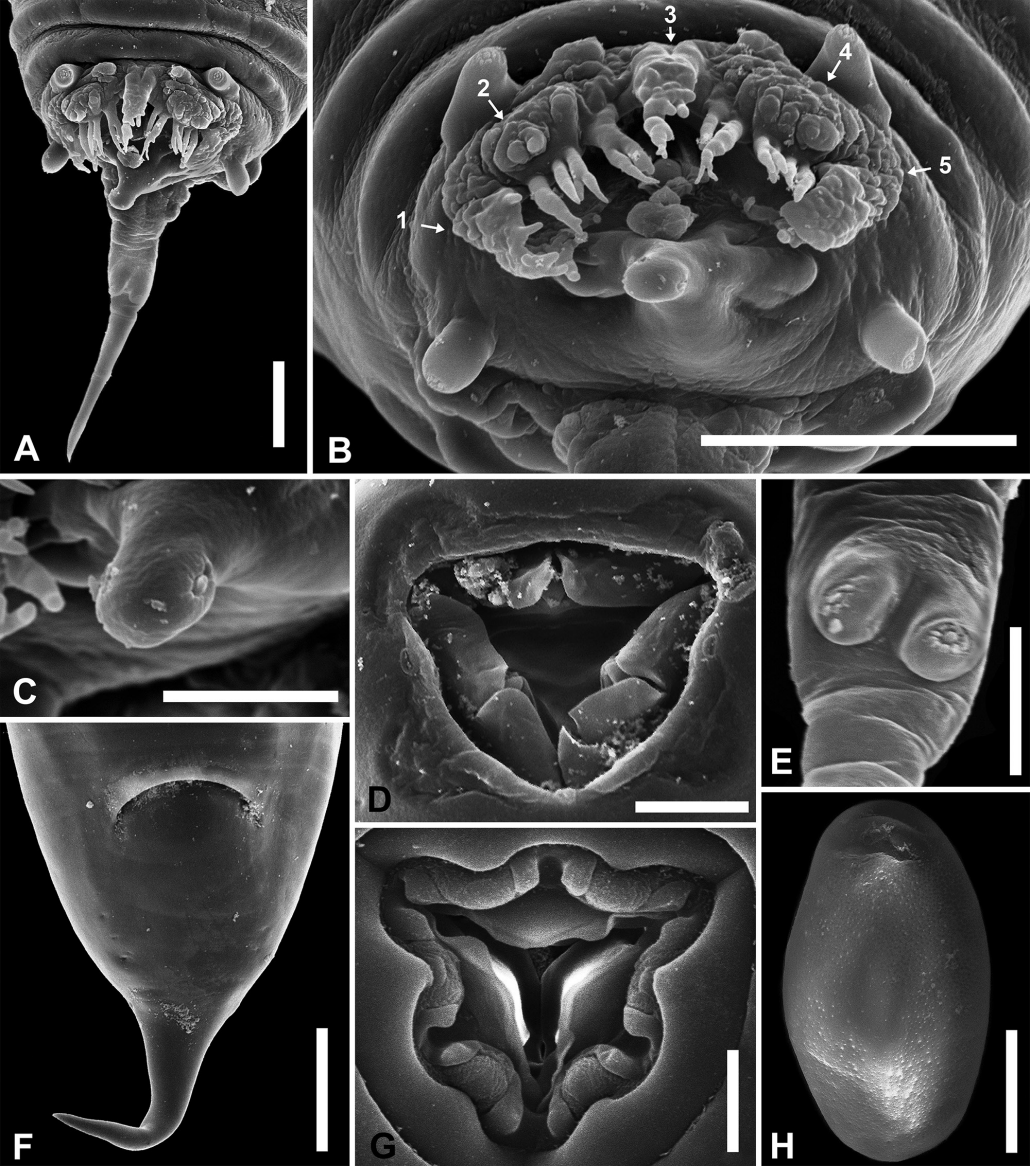
*Parapharyngodon ayotzinapaensis* sp. n. SEM microphotographs. **A** Male, ventral view of posterior end **B** Male, ventral view of posterior end showing cloacal lip and papillae (1-5 outgrowths ornamentation disposition) **C** Male, posterior end of post cloacal lip showing right papillae **D** Male, oral opening **E** Male, pair of papillae at caudal filament **F** Female, ventral view of posterior end **G** Female, oral opening **H** Egg. Scale bars = (**A**) 0.025 mm, (**B**) 0.04 mm, (**C, D, E, F, G**) 0.01 mm, (**H**) 0.02 mm.

Distinctly truncate posterior end (Fig. 1E), total body length 2.72–3.92 (3.458 ± 0.458, n = 5), maximum width 0.6–1.88 (1 ± 0.505, n = 5) at middle region. Cuticle with wide transverse striae 0.05–0.08 (0.062 ± 0.011, n = 5) maximum width at middle region. Triangular oral opening surrounded by three simple lips at whose internal bases are located three transverse bilobed plates, ventrolateral lips with one amphid each one (Fig. 2D). Esophagus total length 0.7–0.84 (0.79 ± 0.054, n = 5) and maximum width 0.04–0.06 (0.05 ± 0.01, n = 5), esophageal bulb length 0.12–0.17 (0.148 ± 0.023, n = 5) and width 0.13–0.16 (0.146 ± 0.013, n = 5). Nerve ring and excretory pore 0.1–0.24 (0.172 ± 0.064, n = 4) and 0.74–1.39 (1.046 ± 0.237, n = 5) from anterior end, respectively. Testis extends from middle body region to level of anterior end of intestine. Lateral alae start abruptly from level of the beginning of third caudal region of body (Fig. 1E). The left one at 2.24–3.01 (2.573 ± 0.395, n = 3), and the right one at 2.31–2.88 (2.565 ± 0.253, n = 4) from anterior end, both with a maximum width of 0.06–0.07 (0.062 ± 0.005, n = 4); extending to posterior end of body, the left one at 0.02–0.07 (0.046 ± 0.025, n = 3) and the right one at 0.03–0.09 (0.056 ± 0.023, n = 5) from the base of corresponding paracloacal papillae. Four pairs of caudal papillae distributed as follows: one precloacal pedunculate, one paracloacal pedunculate, one lateral sessile at the central lobe apex of postcloacal lip and one mammilliform at 0.06–0.07 (0.065 ± 0.006, n = 4) from posterior end of caudal filament (Fig. 2A and B). Top of pedunculate and mammilliform papillae in a rosette-like structure (Fig. 2A, B and E). Echinate precloacal lip with rough appearance, at base of posteriorly directed finger-like ornamentation, which vary in simple or bifurcate outgrowths disposition (Figs 1B; 2B). In SEM pictures morphology is distinguished as follows: cloacal lip rise up from anterior cloacal edge, at both end sides it has a rough thickening region longer that remainder of lip (1 and 5 in Fig. 2B), adjacent to these, at both sides of lip and towards central axis of body, there are finger-like outgrowths that start from rough regions (2 and 4 in Fig. 2B). On the central axis of body is located a rough region (3 in Fig. 2B) upon which are situated finger-like outgrowths, at each side of this region other finger-like outgrowths are situated. Posterior cloacal lip is divided into three lobes, the middle one is more developed than lateral lobes, it is 0.03–0.05 (0.04 ± 0.008, n=4) length and has a pair of tiny simple papillae (Fig. 2C). Caudal filament 0.09–0.1 (0.093 ± 0.006, n = 3) length. Phasmids situated on caudal filament base laterally. Spicule length 0.11–0.13 (0.12 ± 0.01, n = 3) 3.614% of body length, distal end obtuse and thinnest than proximal (Fig. 1D).

##### Description of female.

Round anterior end and conical posterior end (Fig. 1A), total body length 6.41–9.32 (7.45 ± 1.149, n = 8) and maximum width 0.76–1.26 (0.94 ± 0.189, n = 8) at middle body region. Cuticle with wide transverse striae 0.07–0.09 (0.08 ± 0.01, n = 8) maximum width at esophageal bulb level. Triangular oral opening surrounded by three bilobed lips whose internal base are located three transverse complete plates, ventrolateral lips with one amphid each one located at dorsal lobe (Fig. 2F). Esophagus total length 1.17–1.35 (1.269 ± 0.073, n = 8) and maximum width 0.07–0.09 (0.076 ± 0.007, n = 8), esophageal bulb length 0.15–0.21 (0.177 ± 0.019, n = 7) and width 0.21–0.28 (0.24 ± 0.024, n = 7). Nerve ring and excretory pore 0.15–0.21 (0.181 ± 0.023, n = 8) and 1.71–2.4 (1.937 ± 0.237, n = 8) from anterior end, respectively. Sclerotized vulva at 2.95–4.13 (3.45 ± 0.396, n = 8) from anterior end. Vagina transversely directed and posteriorly flexed to posterior region of body. Didelphic, prodelphic, ovaries reach esophagus region coiling around prebulbar esophagus. Uterus reach caudal region in gravid individuals. Anus 0.4–0.48 (0.435 ± 0.039, n = 6) from posterior end. Phasmids 0.14–0.25 (0.204 ± 0.042, n = 8) from posterior end, located laterally at the base of the conical tail (Fig. 2F). Tail 0.2–0.26 (0.237 ± 0.026, n = 8) length. Eggs containing embryo in early stage of cleavage, oval, without alae, asymmetric, slightly flattened on one side and convex on the other side in lateral view, 0.07–0.09 (0.08 ± 0.006, n = 16) length by 0.03–0.05 (0.04 ± 0.005, n=16) width, shell with pores that cross the uppermost layer, radial striations in lateral view, subpolar operculum without pores (Figs 1C; 2H).

##### Distribution.

Técpan de Galeana, Guerrero, Mexico (coordinates, see Table 1; elevation 22 m).

##### Biology.

Nematode species parasite of the intestine of *Sceloporus pyrocephalus* Cope, collected on July 6, 2005.

##### Remarks.

*Parapharyngodon ayotzinapaensis* sp. n. is the 79^th^ species assigned to *Parapharyngodon* and the 48^th^ valid species of the genus (Velarde-Aguilar et al. 2015; Bursey and Goldberg 2015). It is distinguished from the other *Parapharyngodon* species by a combination of characters including the possession of 4 pairs of caudal papillae, an echinate anterior cloacal lip, lateral alae covering the last third of body, spicule length representing 3.614% of body length, prebulbar ovaries coiling around prebulbar esophagus and eggs with punctuated shell and without alae. Of the 47 valid species before this study, 12 species have cloacal papillar arrangement similar to *Parapharyngodon ayotzinapaensis* (four pairs of caudal papillae: one precloacal, one paracloacal, one at postcloacal lip and one at caudal filament), as well as echinate precloacal lip and lateral alae, namely: *Parapharyngodon adramitana* Adamson & Nasher, 1984, *Parapharyngodon almoriensis* (Karve, 1949) Freitas, 1957, *Parapharyngodon anomalus* Hobbs, 1996, *Parapharyngodon brevicaudatus* Bogdanov & Markov,1955, *Parapharyngodon colonensis* Bursey, Goldberg & Telford, 2007, *Parapharyngodon dolgieli* (Markov & Bogdanov, 1965) Adamson & Nasher, 1984, *Parapharyngodon echinatus* (Rudolphi, 1819) Freitas, 1957, *Parapharyngodon grenadaensis* Bursey, Drake, Cole, Sterner, Pinckney & Zieger, 2013, *Parapharyngodon margaritiferi* Hering-Hagenbeck, 2001, *Parapharyngodon meridionalis* (Chabaud & Brygoo, 1962) Adamson, 1981, *Parapharyngodon micipsae* (Seurat, 1917) Freitas, 1957 and *Parapharyngodon rousseti* (Tcheprakoff, 1966) Adamson & Nasher, 1984. Only 2 of these species (*Parapharyngodon grenadaensis* and *Parapharyngodon colonensis*) shares with the new species the egg shell punctuated and prebulbar ovaries in females; however, these two species differ in several morphological features with *Parapharyngodon ayotzinapaensis* sp. n.: *Parapharyngodon grenadaensis* and *Parapharyngodon colonensis* have three bilobed lips, which are simple in *Parapharyngodon ayotzinapaensis*; lateral alae extension covers from nerve ring level to precloacal papillae in *Parapharyngodon grenadaensis* and *Parapharyngodon colonensis*, meanwhile in *Parapharyngodon ayotzinapaensis* begins abruptly at last third of caudal region ending before paracloacal papillae. Finally, spicule length/body length ratio is greater in *Parapharyngodon grenadaensis* (4.488%) and *Parapharyngodon colonensis* (3.765%) than in *Parapharyngodon ayotzinapaensis* (3.614%). Therefore, we consider *Parapharyngodon ayotzinapaensis* to represent a new species, the 10th recorded in Mexico (Bursey and Goldberg 2005, Bursey et al. 2013, Velarde-Aguilar et al. 2015, Bursey and Goldberg 2015).

#### Parapharyngodon tikuinii sp. n.

Taxon classificationAnimaliaOxyuridaPharyngodonidae

http://zoobank.org/C9D2FD3C-F12E-47A8-A755-E06EEEE9502C

Figs 3A–D ; 4A–F

##### Type material.

Holotype, male, CNHE 9439. Allotype, female, CNHE 9440. Paratypes (8 males, 6 females), CNHE 9441–9447.

##### Etymology.

The species is named after the Purepecha word “tikuini” which means lizard, referring to the host (Lathrop 1973).

##### Diagnosis.

Fusiform and robust nematodes, males smaller than females. Cuticle with thin transverse striations constant in width along the whole body except tail. Triangular oral opening surrounded by three lips simple in males and bilobed in females, in both sexes ventrolateral lips with an amphid each one. Within buccal cavity, both sexes have three transverse plates, bilobed in males and complete in females. Esophageal bulb with sclerotized apparatus. Excretory pore evident, it is located posterior to esophageal bulb-intestine junction. A vesicular body surrounds excretory duct. Males with lateral alae covering almost of body length, females lacking lateral alae. Males without caudal alae. Four pairs of caudal papillae. Caudal filament subterminal and directed dorsally in males. Females with a conical posterior end. Vulva located at middle region. Eggs without alae with punctuated shell and a subpolar operculum, embryo in early stage of cleavage.

##### Description of male.

**Figure 3.**
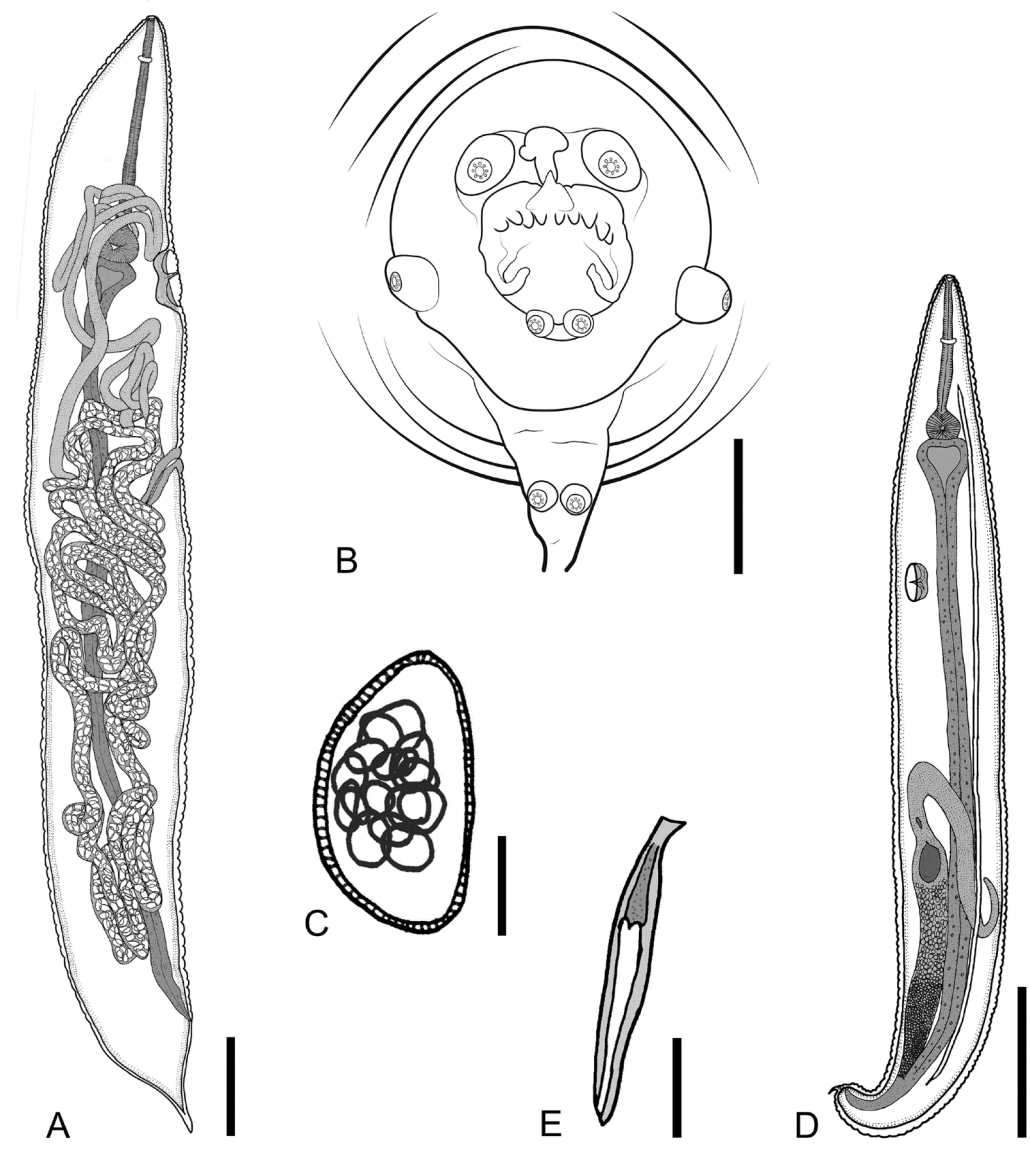
*Parapharyngodon tikuinii* sp. n. **A** Gravid female, entire, lateral view **B** Male, caudal end, ventral view **C** Egg, lateral view **D** Male, entire, lateral view **E** Spicule. Scale bars = (**A**) 0.5 mm, (**B**) 0.02 mm, (**C**) 0.025 mm, (**D**) 0.5 mm, (**E**) 0.0125 mm.

**Figure 4.**
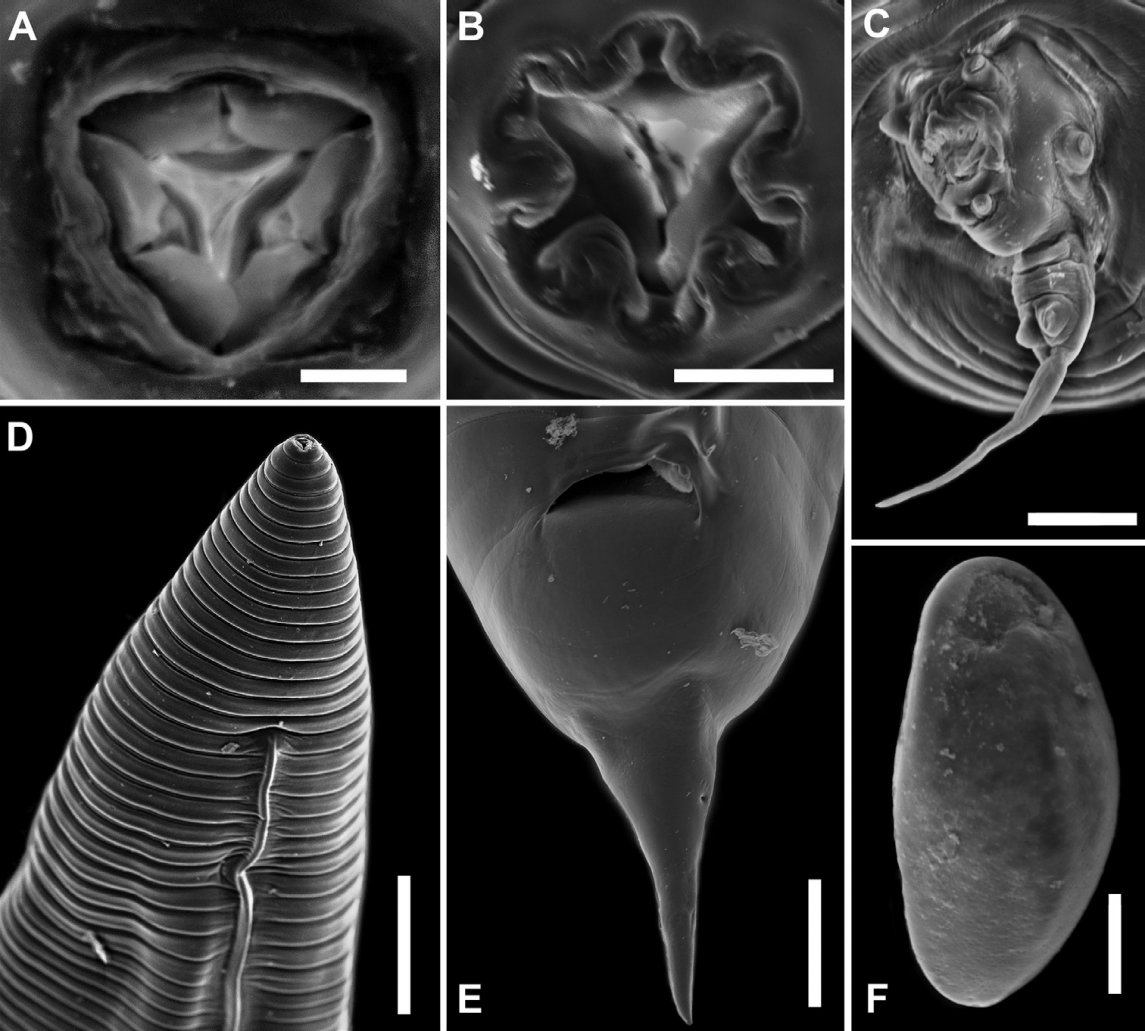
*Parapharyngodon tikuinii* sp. n. SEM microphotographs. **A** Male, oral opening **B** Female, oral opening **C** Male, posterior end, ventrolateral view **D** Male, anterior end showing lateral alae, lateral view **E** Female, posterior end, ventral view **F** Egg. Scale bars = (**A**) 0.05 mm, (**B**) 0.015 mm, (**C**) 0.025 mm, (**D**) 0.1 mm, (**E**) 0.1 mm, (**F**) 0.015 mm.

Truncated at posterior end (Fig. 3D), total body length 1.9–3.575 (2.62 ± 0.5, n = 9) and maximum width 0.237–0.475 (0.326 ± 0.09, n = 6) at excretory pore level. Cuticle with transverse striae 0.015–0.03 (0.02 ± 0.005, n = 7) maximum width at esophageal bulb level. Triangular oral opening surrounded by three simple lips, at whose internal bases are located three transverse bilobed plates, ventrolateral lips with one amphid each one (Fig. 4A). Esophagus total length 0.342–0.53 (0.416 ± 0.056, n = 8) and maximum width 0.022–0.04 (0.0325 ± 0.006, n = 8), esophagus bulb length 0.075–0.105 (0.09 ± 0.01, n = 8) and width 0.08–0.11 (0.09 ± 0.012, n = 8). Nerve ring and excretory pore at 0.1–0.19 (0.147 ± 0.038, n = 6) and 0.71–1.02 (0.857 ± 0.11, n = 6) from anterior end, respectively. Testis extends from middle body region to level of anterior end of intestine. Hyaline lateral alae start at anterior region between nerve ring and excretory pore at 0.25–0.45 (0.334 ± 0.07, n = 6) from anterior end, with a maximum width of 0.06–0.075 (0.065 ± 0.008, n = 7) and extending to posterior end of body terminating abruptly before cloacal region at 0.12–0.435 (0.263 ± 0.11, n = 7) from the base of corresponding paracloacal papillae (Figs 3D; 4D). Four pairs of caudal rosette papillae as follows: one ventral precloacal pedunculated, one lateral postcloacal pedunculated, one postcloacal mammilliform on the posterior base of postcloacal lip, and one mammilliform on the caudal filament (Figs 3B; 4C). Anterior cloacal lip echinate, with symmetrical ornamentation consisting of a smooth outgrowth with V-form at each side of cloacal opening, in the middle of the lip are located small equidistant simple finger-like outgrowths which vary in number (Figs 3B; 4C). Thick and smooth posterior cloacal lip, with a cuticular outgrowth at its base. This structure has two papillae at the top. Phasmids located at caudal filament base, whose length is 0.045–0.0775 (0.064 ± 0.013, n = 7). Spicule length 0.07–0.11 (0.093 ± 0.014, n = 7), 3.287% of total body length, proximal end wider than distal obtuse tip (Fig. 3E).

##### Description of female.

Round anterior end, conical posterior end (Fig. 3A). Total body length 4.47–7.57 (6 ± 1.213, n = 7) and maximum width 1.04–1.3 (1.18 ± 0.09, n = 7) at middle body level. Cuticle with transverse striae 0.05–0.08 (0.06 ± 0.01, n = 7) maximum width. Triangular oral opening surrounded by three bilobed lips with an amphid located at dorsal lobe of each ventrolateral lip, at whose internal bases are located three transverse complete plates (Fig. 4B). Esophagus length 1.25–1.56 (1.367 ± 0.134, n = 4) and maximum width 0.07–0.085 (0.075 ± 0.005, n = 6), esophageal bulb length 0.187–0.257 (0.219 ± 0.029, n = 4) by 0.21–0.25 (0.236 ± 0.02, n = 4) width. Nerve ring and excretory pore at 0.15–0.28 (0.21 ± 0.065, n = 3) and 1.17–1.97 (1.658 ± 0.31, n = 5) from anterior end, respectively. Sclerotized vulva at 1.33–3.32 (2.61 ± 0.716, n = 7) from anterior end. Vagina transversely directed and posteriorly flexed to posterior region of body (Fig. 3A). Didelphic, prodelphic, ovaries reach esophagus region coiling around prebulbar esophagus. Uterus reaching caudal region in gravid individuals. Anus 0.44–0.64 (0.566 ± 0.065, n = 6) from posterior end. Phasmids 0.09–0.28 (0.182 ± 0.095, n = 3) from posterior end (Fig. 4E), located laterally at the base of the conical tail. Tail 0.182–0.285 (0.232 ± 0.04, n = 7) length. Eggs containing embryo in early stage of cleavage, oval, without alae, asymmetric, slightly flattened on one side and convex on the other side in lateral view, 0.067–0.087 (0.078 ± 0.006, n = 13) length by 0.02–0.05 (0.04 ± 0.008, n = 12) width, shell egg with pores that cross the uppermost layer, radial striations in lateral view, subpolar operculum smooth without pores (Figs 3C; 4F).

##### Distribution.

Gabriel Zamora, Michoacán, Mexico (19°10'35"N, 102°03'48"W, elevation 752 m).

##### Biology.

Nematode species parasite of the intestine of *Sceloporus pyrocephalus* Cope, collected on June 21, 2004.

## Remarks

*Parapharyngodon tikuinii* sp. n. is the 80^th^ species assigned to *Parapharyngodon* and the 49^th^ valid species of the genus. It is characterized by the presence of a cuticular outgrowth at base of posterior cloacal lip. In addition, the following composition of traits allow us to differentiate the new species described herein: four pairs of caudal papillae, an echinate anterior cloacal lip, lateral alae covering almost the length of the body, spicule length 3.287% of total body length, prebulbar ovaries coiling around prebulbar esophagus and eggs shell punctuate. Nineteen of the 48 valid species described before (including *Parapharyngodon ayotzinapaensis*), share with *Parapharyngodon tikuinii* the arrangement of caudal papillae (four pairs of caudal papillae: one precloacal, one paracloacal, one at postcloacal lip and one at caudal filament). Thirteen of these species have echinate precloacal lip and lateral alae as the second new species described herein; of these, only *Parapharyngodon grenadaensis*, *Parapharyngodon colonensis* and *Parapharyngodon ayotzinapaensis* share the presence of prebulbar ovaries and a punctuate egg shell with *Parapharyngodon tikuinii*. Nonetheless, *Parapharyngodon tikuinii* differs from *Parapharyngodon grenadaensis*, *Parapharyngodon colonensis* and *Parapharyngodon ayotzinapaensis* in the lateral alae extension (which start at level of nerve ring and end at level of precloacal papillae in these three species, whereas in *Parapharyngodon tikuinii* lateral alae cover exclusively the last portion of the body); in addition, spicule length-total body length ratio is greater in *Parapharyngodon grenadaensis* (4.488%), *Parapharyngodon colonensis* (3.765%) and *Parapharyngodon ayotzinapaensis* (3.614%) than in *Parapharyngodon tikuinii* (3.287%) (Bursey and Goldberg 2005, Bursey et al. 2013, Velarde-Aguilar et al. 2015, Bursey and Goldberg 2015). Finally, males of *Parapharyngodon grenadaensis* and *Parapharyngodon colonensis* have three bilobed lips which are simple in *Parapharyngodon tikuinii*. Consequently, *Parapharyngodon tikuinii* is proposed as new species for the genus and the 11th recorded in Mexico.

## Discussion

*Parapharyngodon* includes species parasitizing ectothermic vertebrates (mainly reptiles), few species of amphibians [*Rhinella marina* Linnaeus (Anura: Bufonidae) parasitized by *Parapharyngodon grenadaensis*, *Phrynohyas venulosa* Laurenti (Anura: Hylidae) parasitized by *Parapharyngodon duniae*, *Onychodactylus japonicus* Houttuyn (Caudata: Hynobiidae) parasitized by *Parapharyngodon japonicus*, *Triprion petasatus* Cope (Anura: Hylidae) parasitized by *Parapharyngodon hylidae*, ﻿and *Diaglena spatulata* Günther (Anura: Hylidae) parasitized by *ParapharyngodonParapharyngodon chamelensis*] and one ancestral mammal species [*Tachyglossus aculeatus* Shaw (Monotremata: Tachyglossidae) (Irwin and Raharison 2009)]. Only two species of *Parapharyngodon* have been described as parasites of phrynosomatid lizards: *Parapharyngodon grismeri* and *Parapharyngodon iguanae* in *Petrosaurus repens* Van Denburgh and *Petrosaurus mearnsi* Stejneger, respectively (Paredes-León et al. 2008, Velarde-Aguilar et al. 2015). In this study, we describe two additional species infecting phrynosomatid lizards: *Parapharyngodon ayotzinapaenis* and *Parapharyngodon tikuinii*.

Some authors had emphasized the relationship between food habits and composition of helminths richness in reptiles (Martin et al. 2005, Pereira et al. 2013, Roca 1999, Roca et al. 2005). In this sense, Petter (1966) and Petter and Quentin (1976) recognized an evolutionary trend within Pharyngodonidae and distinguished two groups of genera: 1) Parasites of herbivorous iguanids and testudines, and 2) Parasites of omnivorous and insectivorous reptiles. In Mexico, nine pharyngodonid genera have been registered, five in herbivorous iguanids and testudines (*Ozolaimus* Dujardin, 1845; *Tachygonetria* Wedl, 1862; *Alaeuris* Thapar, 1925; *Thaparia* Ortlepp, 1933; *Gopheruris* Petter & Douglas, 1976), and four in omnivorous and insectivorous reptiles (*Parapharyngodon*; *Pharyngodon*, Diesing, 1861; *Spauligodon* Skrjabin, Schikhobalova & Lagodovskaja, 1960, and *Skrjabinodon* Inglis, 1968). In this sense, the presence of *Parapharyngodon* species infecting *Sceloporus pyrocephalus* confirm indirectly the feeding habits observed in this lizard, which is considered an omnivorous and insectivorous species by Alvarado-Diaz et al. (2009). On the other hand, there are scarce studies focused on evolutionary history of Pharyngodonidae and only include few genera from the same region (Jorge et al. 2011, Jorge et al. 2012) and some species parasitic in herbivores (Bouamer and Morand 2003). This information is not enough to attempt to relate the trend observed among pharyngodonids to parasitize hosts based on its different feeding habits with their evolutionary history. Robust analyses based on molecular and morphological information about the relationship of pharyngodonid species and their hosts will allow analysis of the patterns and process involved in their evolutionary history.

### Key to Mexican species of *Parapharyngodon*

**Table:** 

1	Lateral alae present	**2**
–	Lateral alae absent	***Parapharyngodon californiensis***
2	Lateral alae covering almost total body length	**3**
–	Lateral alae confined to posterior region of the body	**9**
3	With three pairs of caudal papillae	**4**
–	More than three pairs of caudal papillae	**7**
4	Lateral alae start at level of esophageal bulb	***Parapharyngodon guerreroensis***
–	Lateral alae start at half the length of the esophagus	**5**
5	Lateral alae extend to level of the first pair of precloacal papillae	***Parapharyngodon alvarengai***
–	Lateral alae extend to four fifth of the length body	**6**
6	Gubernaculum present	***Parapharyngodon hylidae***
–	Gubernaculum absent	***Parapharyngodon maestro***
7	With four pairs of caudal papillae	***Parapharyngodon tikuinii* sp. n.**
–	With three pairs of caudal papillae and one extra papilla	**8**
8	Protuberance in posterior cloacal lip present	***Parapharyngodon chamelensis***
–	Protuberance in posterior cloacal lip absent	***Parapharyngodon lamothei***
9	With four pairs of caudal papillae	***Parapharyngodon ayotzinapaensis* sp. n.**
–	With 3 pairs of caudal papillae	**10**
10	Middle pair of caudal papillae mammilliform	***Parapharyngodon grismeri***
–	Middle pair of caudal papillae digitiform	***Parapharyngodon iguanae***

## Supplementary Material

XML Treatment for Parapharyngodon ayotzinapaensis

XML Treatment for Parapharyngodon tikuinii
